# Cost-Effectiveness of Pharmacotherapy for the Treatment of Obesity in Adolescents

**DOI:** 10.1001/jamanetworkopen.2023.29178

**Published:** 2023-08-31

**Authors:** Francesca Lim, Brandon K. Bellows, Sarah Xinhui Tan, Zainab Aziz, Jennifer A. Woo Baidal, Aaron S. Kelly, Chin Hur

**Affiliations:** 1 Department of Medicine, Columbia University Irving Medical Center, New York, New York; 2 Department of Environmental Health Sciences, Columbia University Mailman School of Public Health, New York, New York; 3 Department of Pediatrics, Columbia University Irving Medical Center, New York, New York; 4 Department of Pediatrics and Center for Pediatric Obesity Medicine, University of Minnesota Medical School, Minneapolis

## Abstract

**Importance:**

Antiobesity pharmacotherapy is recommended for adolescents ages 12 years and older with obesity. Several medications have been approved by the US Food and Drug Administration for adolescent use, but the most cost-effective medication remains unclear.

**Objective:**

To estimate the cost-effectiveness of lifestyle counseling alone and as adjunct to liraglutide, mid-dose phentermine and topiramate (7.5 mg phentermine and 46 mg topiramate), top-dose phentermine and topiramate (15 mg phentermine and 92 mg topiramate), or semaglutide among adolescent patients with obesity.

**Design, Setting, and Participants:**

This economic evaluation used a microsimulation model to project health and cost outcomes of lifestyle counseling alone and adjunct to liraglutide, mid-dose phentermine and topiramate, top-dose phentermine and topiramate, or semaglutide over 13 months, 2 years, and 5 years among a hypothetical cohort of 100 000 adolescents with obesity, defined as an initial body mass index (BMI; calculated as weight in kilograms divided by height in meters squared) of 37. Model inputs were derived from clinical trials, published literature, and national sources. Data were analyzed from April 2022 to July 2023.

**Exposures:**

Lifestyle counseling alone and as adjunct to liraglutide, mid-dose phentermine and topiramate, top-dose phentermine and topiramate, or semaglutide.

**Main Outcomes and Measures:**

The main outcome was quality-adjusted life years (QALYs), costs (2022 US dollars), and incremental cost-effectiveness ratios (ICERs), with future costs and QALYs discounted 3.0% annually. A strategy was considered cost-effective if the ICER was less than $100 000 per QALY gained. The preferred strategy was determined as the strategy with the greatest increase in QALYs while being cost-effective. One-way and probabilistic sensitivity analyses were used to assess parameter uncertainty.

**Results:**

The model simulated 100 000 adolescents at age 15 with an initial BMI of 37, of whom 58 000 (58%) were female. At 13 months and 2 years, lifestyle counseling was estimated to be the preferred strategy. At 5 years, top-dose phentermine and topiramate was projected to be the preferred strategy with an ICER of $56 876 per QALY gained vs lifestyle counseling. Semaglutide was projected to yield the most QALYs, but with an unfavorable ICER of $1.1 million per QALY gained compared with top-dose phentermine and topiramate. Model results were most sensitive to utility of weight reduction and weight loss of lifestyle counseling and top-dose phentermine and topiramate.

**Conclusions and Relevance:**

In this economic evaluation of pharmacotherapy for adolescents with obesity, top-dose phentermine and topiramate as adjunct to lifestyle counseling was estimated to be cost-effective after 5 years. Long-term clinical trials in adolescents are needed to fully evaluate the outcomes of pharmacotherapy, especially into adulthood.

## Key Points

**Question:**

Compared with lifestyle counseling alone, is liraglutide, mid-dose phentermine and topiramate, top-dose phentermine and topiramate, or semaglutide adjunct to lifestyle counseling cost-effective in treating obesity among adolescents in a simulated cohort?

**Findings:**

In this economic evaluation including 100 000 simulated adolescents, no pharmacotherapy was estimated to be cost-effective after 13 months and 2 years of treatment. After 5 years, top-dose phentermine and topiramate was projected to be cost-effective, with an incremental cost-effectiveness ratio of $56 876 per quality-adjusted life year gained.

**Meaning:**

This economic evaluation found that top-dose phentermine and topiramate adjunct to lifestyle counseling was cost-effective for the treatment of obesity in adolescent patients after 5 years.

## Teaser

This economic evaluation estimates the cost-effectiveness of lifestyle counseling alone and as adjunct to liraglutide, mid-dose phentermine and topiramate, maximum-dose phentermine and topiramate, or semaglutide among adolescent patients with obesity.

## Introduction

Adolescent obesity, defined as body mass index (BMI; calculated as weight in kilograms divided by height in meters squared) greater than or equal to the age- and sex-specific 95th percentile of the 2000 US Centers for Disease Control and Prevention (CDC) growth charts,^1^ is a complex, chronic disease associated with increased comorbidity that extends into adulthood.^2,3,4,5^ The prevalence of adolescent obesity in the US has increased for nearly 2 decades, currently affecting more than 1 in 5 adolescents.^6^ Intensive health behavior and lifestyle intervention (eg, dietary counseling and physical activity) is recommended as first-line treatment,^7,8^ but BMI reduction is often small, and high attrition rates are usually observed.^9,10,11^ In 2023, the American Academy of Pediatrics (AAP) recommended antiobesity medications (AOMs) be offered as an adjunct to intensive health behavior and lifestyle counseling in adolescents with obesity ages 12 years and older.^7^

Significant placebo-subtracted BMI reductions from baseline to approximately 1 year have been seen with pharmacologic treatment of obesity in clinical trials of adolescents ages 12 years to younger than 18 years, with reductions of 4.6% reported for liraglutide,^12^ 8.1% reported for mid-dose phentermine and topiramate (ie, 7.5 mg phentermine and 46 mg topiramate daily),^13^ 10.4% reported for top-dose phentermine and topiramate (ie, 15 mg phentermine and 92 mg topiramate daily),^13^ and 16.7% reported for semaglutide.^14^ Subsequently, the US Food and Drug Administration (FDA) has approved liraglutide, phentermine and topiramate, and semaglutide for treatment of obesity in adolescents ages 12 years and older. However, these are proprietary medications and have annual costs ranging from $1100 to $15 000. Estimating the cost-effectiveness of these adjunctive AOMs may help guide uptake and pricing.

The purpose of this study was to estimate the costs, quality-adjusted life-years (QALYs), and cost-effectiveness of lifestyle counseling alone and adjunct to liraglutide, mid-dose phentermine and topiramate, top-dose phentermine and topiramate, or semaglutide to treat US adolescents with obesity over 13 months and estimate projections out to five years.

## Methods

This economic evaluation used computer-based modeling and is not considered human participants research at Columbia University; therefore, neither institutional review board approval nor informed consent were sought. Reporting of this study followed the Consolidated Health Economic Evaluation Reporting Standards (CHEERS) reporting guideline. The Criteria for Health Economic Quality Evaluation tool was referenced for methods and reporting quality.^15^

### Model Overview

We adapted a previously published patient-level microsimulation model for the treatment of obesity in adults to assess the cost-effectiveness of lifestyle counseling alone and adjunct to liraglutide (3 mg daily), mid-dose phentermine and topiramate, top-dose phentermine and topiramate, or semaglutide (2.4 mg weekly) in adolescents with obesity.^16^ The model simulated a hypothetical cohort of 100 000 adolescents with baseline characteristics similar to participants in clinical trials: age 15 years, 58 000 (58%) female, and BMI of 37.^12,13,14^ While clinical trials stated their eligibility as BMI at the 95th percentile or higher according to sex- and age-specific growth charts, the average participant had severe obesity, with a mean BMI of 120% or more of the 95th percentile and/or BMI of at least 35.^11^ Cost-effectiveness was assessed at 13 months (ie, the most common treatment period in clinical trials), 2 years, and 5 years (eMethods in Supplement 1). We did not make projections beyond 5 years due to lack of long-term data.

**Figure 1.**
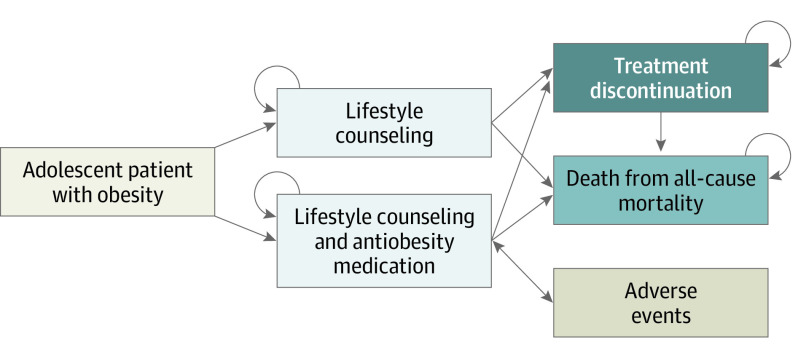
Microsimulation Model Overview All patients began in the microsimulation model receiving treatment, either lifestyle counseling alone or adjunct to an antiobesity medication, ie, liraglutide, mid-dose phentermine and topiramate (7.5 mg phentermine and 46 mg topiramate), top-dose phentermine and topiramate (15 mg phentermine and 92 mg topiramate), or semaglutide. Every month of the simulation, patients could either continue receiving treatment or permanently discontinue treatment. Patients receiving lifestyle counseling adjunct to an antiobesity medication could experience an adverse event every month. Monthly change in body mass index was dependent on the specific treatment strategy, and all patients experienced weight gain after discontinuing any treatment. Death was determined from age- and sex-specific mortality rates.

Each month of the simulation, individuals could remain on treatment, permanently discontinue treatment, or die (Figure 1). Adverse events were incorporated for AOM treatment. All-cause mortality rates were derived from BMI-specific life tables (eTable 1 in Supplement 1).^16,17^ Other model inputs were derived from clinical trials, published literature, and national sources.^12,13,14,18,19,20,21,22,23,24,25,26,27,28,29,30,31,32,33,34,35^

### Strategies

Lifestyle counseling consisted of counseling on healthy nutrition, including a diet aligned with dietary guidelines, and daily moderate-to-high intensity physical activity, with the inclusion of parents or guardians.^12^ Liraglutide and semaglutide are glucagon-like peptide 1 analogues that are administered via subcutaneous injection daily and weekly, respectively, when used for weight management.^12,14,36,37^ Phentermine and topiramate is a fixed-dose combination of immediate-release phentermine and extended-release topiramate administered orally daily for weight management.^13,19,20^ AOMs were administered with lifestyle counseling. Bariatric surgery was not a comparator because it is typically considered for adolescents with a BMI greater than that of our modeled cohort.^38^

### BMI Change

A natural BMI trajectory estimating BMI change without treatment was derived from extended BMI-for-age growth charts from the CDC.^39^ At age 15 years, a BMI of 37.0 is greater than 120% of the 95th percentile for girls and boys. We assumed no change in the BMI-for-age percentile occurred, resulting in a BMI of 42.5 after 5 years without treatment.

Patients receiving treatment experienced BMI reduction relative to their natural BMI trajectory. When an individual discontinued treatment, they regained BMI at an increased rate until they returned to their expected natural BMI. The number of months required to return to their natural BMI was derived from the liraglutide trial by Kelly et al^12^ and was dependent on how long treatment was received (eMethods and eTable 2 in Supplement 1). BMI changes for each strategy were modeled from clinical trials.^12,13,14^ Lifestyle counseling alone was based on the placebo group of the liraglutide trial by Kelly et al.^12^ BMI reduction while receiving treatment was calibrated to reproduce the intention-to-treat values for relative change in BMI from baseline, accounting for the proportion of individuals discontinuing treatment (eMethods and eTable 3 in Supplement 1).^12,13,14^ Calibration was validated by comparing the model output of relative BMI change from baseline with reported intention-to-treat values.

Due to lack of long-term follow-up data, we made several assumptions to extrapolate BMI beyond the treatment period of the adolescent trials. For lifestyle counseling, individuals receiving treatment maintained the same relative BMI reduction compared with their natural BMI trajectory achieved at 13 months (eMethods in Supplement 1). For AOMs, BMI changes were derived from adult clinical trials.^18,19,20,21,22^ In adults, weight changes from baseline to approximately 1 and 2 years of treatment were converted to BMI changes using mean baseline weight and BMI (eTable 4 in Supplement 1). We assumed the same relative BMI change from 1 to 2 years in adults applied to adolescents (eMethods and eTable 3 in Supplement 1). Patients who continued to receive treatment for at least 2 years did not experience further BMI reduction and maintained the same relative change compared with their natural BMI trajectory achieved at 2 years.

### Treatment Discontinuation

The probability of discontinuing treatment was informed by adolescent clinical trials.^12,13,14^ The probability of discontinuing treatment beyond the treatment period was derived from adult trials (eMethods and eTable 5 in Supplement 1).^18,19,20,21,22^ All individuals who were still receiving treatment at 2 years were assumed to continue treatment until 5 years. Patients who discontinued use of AOMs also discontinued lifestyle counseling.

### Adverse Events

Gastrointestinal (eg, nausea, vomiting, diarrhea), psychiatric (eg, depression, anxiety), and severe adverse events (AEs) were incorporated in the model as they were the main 3 types of AEs reported in adolescent clinical trials (eMethods in Supplement 1).^12,13,14^ The probability of AEs was not projected beyond the duration of the trials. However, most events occurred within the first several weeks of treatment.

### Quality-of-Life Adjustments and Costs

The initial utility of severe obesity in adolescents was obtained from published literature.^23^ An increase in utility was incorporated per 1-unit decrease in BMI.^24^ Short-term disutilities from AEs were also included (eMethods in Supplement 1).^25,26,27^ Utilities were derived from a patient perspective.

We adopted a health care sector and limited societal perspective (eMethods in Supplement 1). An hour of time was valued to be the mean hourly wage from the US Bureau of Labor Statistics, $32.^40^ The cost of lifestyle counseling was estimated from a family-based intervention for pediatric obesity (eMethods in Supplement 1).^28^ Upper and lower bounds were estimated from similar interventions.^29,41^ The base case monthly cost of AOMs was determined from the Center for Medicare and Medicaid Services National Average Drug Acquisition Cost.^30^ Upper and lower bounds of estimates were the mean wholesale price from Micromedex Red Book and the lowest price from the Department of Veterans Affairs Federal Supply Schedule, respectively (eMethods in Supplement 1).^31,32^ A cost of 2 physician visits was added to the first month of AOM treatment to account for extra visits for initiation.^16,33,42^ Patients who experienced a nonsevere AE received the cost of 1 physician visit. Costs of severe AEs were estimated from the costs of severe drug AEs.^34^ Patients who discontinued treatment did not accrue further costs. Total medical care costs for obesity were estimated from nationally representative data (eMethods in Supplement 1).^35^ All costs are reported in 2022 US dollars and were adjusted using the health care component of the Personal Consumption Expenditures price index.^43^ Model inputs are detailed in Table 1. Key model assumptions are summarized in eTable 6 in Supplement 1.

**Table 1.** Microsimulation Model Inputs

Parameter	Value	Range^a^	Distribution^b^	Source
**BMI reduction while receiving treatment, %** ^c^				
Lifestyle counseling, 13 mo	0.2	(−1.1 to 3.1)	NA	Calibrated^d^
Liraglutide				
13 mo	5.5	(3.5 to 7.5)	NA	Calibrated^d^
25 mo	6.3	(4.7 to 7.9)	NA	
Mid-dose phentermine and topiramate				
13 mo	7.0	(3.8 to 10.3)	NA	Calibrated^d^
25 mo	12.2	(7.5 to 16.9)	NA	
Top-dose phentermine and topiramate				
13 mo	11.3	(8.5 to 14.1)	NA	Calibrated^d^
25 mo	19.2	(15.3 to 23.0)	NA	
Semaglutide				
16 mo	17.7	(14.0 to 21.6)	NA	Calibrated^d^
24 mo	19.3	(15.3 to 23.3)	NA	
**Treatment discontinuation, %** ^e^				
Lifestyle counseling				
0-13 mo	20.6	(13.6 to 27.7)	β	Kelly et al,^12^ 2020
14-25 mo	6.7	(1.8 to 11.6)	β	
Liraglutide				
0-13 mo	19.2	(12.3 to 26.1)	β	Kelly et al,^12^ 2020
14-25 mo	16.4	(9.2 to 23.6)	β	Astrup et al,^18^ 2012
Mid-dose phentermine and topiramate				
0-13 mo	29.6	(17.5 to 41.8)	β	Kelly et al,^13^ 2022
14-25 mo	16.1	(4.4 to 27.8)	β	Gadde et al,^19^ 2011; Garvey et al, 2012^20^
Top-dose phentermine and topiramate				
0-13 mo	38.9	(29.9 to 48.0)	β	Kelly et al,^13^ 2022
14-25 mo	20.5	(11.0 to 30.1)	β	Gadde et al,^20^ 2011; Garvey et al,^19^ 2012
Semaglutide				
0-16 mo	10.4	(5.3 to 15.6)	β	Weghuber et al,^14^ 2022
17-24 mo	6.2	(1.9 to 10.5)	β	Wilding et al,^21^ 2021; Garvey et al,^22^ 2022
**AOM-related** AE**s, %**				
Gastrointestinal				
Liraglutide	28.30	NA	NA	Kelly et al,^12^ 2020
Mid-dose phentermine and topiramate	0.00	NA	NA	Kelly et al,^13^ 2022
Top-dose phentermine and topiramate	0.00	NA	NA	Kelly et al,^13^ 2022
Semaglutide	20.00	NA	NA	Weghuber et al,^14^ 2022
Psychiatric				
Liraglutide	0.00	NA	NA	Kelly et al,^12^ 2020
Mid-dose phentermine and topiramate	5.60	NA	NA	Kelly et al,^13^ 2022
Top-dose phentermine and topiramate	7.00	NA	NA	Kelly et al,^13^ 2022
Semaglutide	0.00	NA	NA	Weghuber et al,^14^ 2022
Severe				
Liraglutide	1.60	NA	NA	Kelly et al,^12^ 2020
Mid-dose phentermine and topiramate	0.00	NA	NA	Kelly et al,^13^ 2022
Top-dose phentermine and topiramate	1.80	NA	NA	Kelly et al,^13^ 2022
Semaglutide	2.00	NA	NA	Weghuber et al,^14^ 2022
**Utilities^f^**				
Initial utility for severe obesity alone	0.670	(0.639 to 0.701)	β	Schwimmer et al,^23^ 2003
Increase in utility per 1-unit decrease in BMI	0.004	(0.002 to 0.006)	β	Bairdain and Samnaliev,^24^ 2015
Gastrointestinal AE, AOMs	−0.040	(−0.052 to −0.028)	β	Matza et al,^25^ 2007
Psychiatric AE, AOMs	−0.117	(−0.174 to −0.099)	β	Lynch et al,^26^ 2016
Severe AE, AOMs	−0.100	(−0.130 to −0.080)	β	Bress et al,^27^ 2017
**Costs, 2022 US $**				
Lifestyle counseling, per mo	100	(66 to 114)	γ	Janicke et al,^28^ 2009; Goldfield et al,^41^ 2001; Quattrin et al,^29^ 2017
Liraglutide, per mo	1294	(989 to 1661)	γ	The Centers for Medicare and Medicaid Services National Average Drug Acquisition Cost,^30^ 2022; Department of Veterans Affairs Federal Supply Schedule,^32^ 2022; Micromedex RED BOOK,^31^ 2022
Mid-dose phentermine and topiramate, per mo	179	(93 to 245)	γ	
Top-dose phentermine and topiramate, per mo	191	(120 to 321)	γ	
Semaglutide, per mo	1295	(1044 to 1661)	γ	
Physician visit, AOMs^g^	73	(16 to 191)	γ	The Centers of Medicare and Medicaid Services Preventive Medicine Office Visit Costs,^33^ 2022
Severe AE, AOMs	14 232	(12 956 to 15 507)	γ	Hug et al,^34^ 2012
Annual medical care costs, severe obesity				
Boys	3033	(1607 to 4459)	γ	Biener et al, 2020^35^
Girls	2948	(1685 to 4210)	γ	
Decrease in annual medical care costs per 1-unit BMI reduction				
Boys	94	(29 to 159)	γ	Biener et al,^35^ 2020
Girls	88	(35 to 141)	γ	

Abbreviations: AE, adverse event; AOM, antiobesity medication; BMI, body mass index; NA, not applicable.

^a^ The parameter was not varied in 1-way sensitivity analyses and was kept as its base case value.

^b^ The parameter was not varied in probabilistic sensitivity analyses and was kept as its base case value.

^c^ BMI reduction while receiving treatment is the percentage reduction relative to baseline BMI.

^d^ Calibrated to match the mean intention-to-treat percentage BMI reductions from baseline from clinical trials, accounting for treatment discontinuation (eTable 3 in Supplement 1).

^e^ Treatment discontinuation after 24 months for semaglutide and after 25 months for lifestyle counseling, liraglutide, and phentermine and topiramate was assumed to be zero. Patients who were still receiving treatment at that time point were assumed to continue treatment until 5 years.

^f^ Initial utility for severe obesity and the increase in utility for a 1-unit decrease in BMI were applied across all strategies. A decrease in utility from AEs was only applied to patients receiving AOMs.

^g^ The cost of 2 physician visits were applied to each patient who began treatment with AOMs. The cost of 1 physician visit was applied to each patient who experienced a gastrointestinal or psychiatric AE while using AOMs.

### Statistical Analysis

All analyses were performed using Python statistical software version 3.8.8 (Python Software Foundation). Data analysis was performed from April 2022 to July 2023. Our primary end points were quality-adjusted life years (QALYs), total costs (2022 USD), and incremental cost-effectiveness ratios (ICERs) (eMethods in Supplement 1). Future costs and QALYs were discounted at a rate of 3%. We used a willingness-to-pay (WTP) threshold of $100 000 per QALY to determine cost-effectiveness.^44^ A strategy was considered preferred if it resulted in the greatest increase in QALYs while being cost-effective. Secondary end points included relative BMI change from baseline and relative BMI change from natural BMI trajectory.

To determine the impact of uncertainty of model inputs on results, we performed 1-way and probabilistic sensitivity analyses. One-way sensitivity analyses vary 1 parameter at a time across a range of values while keeping all other inputs at their base-case value (eMethods in Supplement 1). Upper and lower bounds were obtained from minimum and maximum values present in databases or calculated from 95% CIs (eMethods in Supplement 1).

Probabilistic sensitivity analyses were performed by repeatedly sampling model inputs simultaneously from defined probabilistic distributions (eMethods in Supplement 1). We used γ distributions for costs and β distributions for all other parameters. The model was run 1000 times with a cohort of 100 000 patients. We determined the percentage of times each strategy was preferred over a range of different WTP thresholds. To assess uncertainty of mortality estimates, the model was run separately using BMI-specific life tables and unadjusted life tables to compare cost-effectiveness results.

## Results

### Base Case Results

The model simulated 100 000 patients at age 15 years with a BMI of 37, of whom 58 000 (58%) were female. The model replicated intention-to-treat values from randomized clinical trials^12,13,14^ for the relative change in BMI from baseline (eTable 7 in Supplement 1). At 13 months, we projected a relative BMI change from baseline of 0.4% with lifestyle counseling, −4.3% with liraglutide, −4.8% with mid-dose phentermine and topiramate, −7.1% with top-dose phentermine and topiramate, and −13.3% with semaglutide (eFigure 1 and eFigure 2 in Supplement 1). By 5 years, BMI change relative to the natural BMI trajectory (ie, no treatment) was estimated to be −2.2% with lifestyle counseling, −7.4% with liraglutide, −9.5% with mid-dose phentermine and topiramate, −10.8% with top-dose phentermine and topiramate, and −18.5% with semaglutide (eFigure 3 in Supplement 1).

Over 13 months, liraglutide was strictly dominated (ie, cost more and less effective) and mid-dose phentermine and topiramate was extendedly dominated (ie, less effective and higher cost per QALY) compared with top-dose phentermine and topiramate (Table 2 and eFigure 4 in Supplement 1). Top-dose phentermine and topiramate was not cost-effective, with an ICER of $317 010 per QALY gained vs lifestyle counseling. At 2 years, liraglutide and mid-dose phentermine and topiramate were strictly dominated by top-dose phentermine and topiramate. The ICER of top-dose phentermine and topiramate vs lifestyle counseling decreased to $138 045 per QALY gained. By 5 years, top-dose phentermine and topiramate became the preferred strategy, with an ICER of $56 876 per QALY gained vs lifestyle counseling. Over each time horizon, semaglutide was projected to accumulate the most QALYs. However, the ICERs for semaglutide vs top-dose phentermine and topiramate were well above our WTP threshold, ranging from $1.1 to $3.0 million per QALY gained.

**Table 2.** Cost-Effectiveness Results Over Each Time Horizon

Measure	Lifestyle counseling	Liraglutide	Phentermine and topiramate		Semaglutide
			Mid-dose	Top-dose	
**13 mo**					
Costs, mean, $	5052	20 828	7082	7212	21 610
Incremental costs, mean, $	0 [Reference]	15 776	2030	2160	16 557
QALY, mean	0.714	0.718	0.719	0.721	0.726
Incremental QALYs, mean	0 [Reference]	0.003	0.005	0.007	0.012
ICER, $/QALY gained^a^	0 [Reference]	Strictly dominated^b^	Extendedly dominated^c^	317 010	3 029 080
**2 y**					
Costs, mean, $	9043	34 665	11 860	11 641	37 287
Incremental costs, mean, $	0 [Reference]	25 622	2816	2597	28 244
QALY, mean	1.298	1.307	1.312	1.316	1.332
Incremental QALYs, mean	0 [Reference]	0.010	0.014	0.019	0.035
ICER, $/QALY gained^a^	0 [Reference]	Strictly dominated^b^	Strictly dominated^b^	138 045	1 598 963
**5 y**					
Costs, mean ($)	21 732	74 769	26 404	25 086	84 064
Incremental costs, mean, $	0 [Reference]	53 037	4672	3354	62 333
QALY, mean	3.068	3.100	3.117	3.127	3.181
Incremental QALYs, mean	0 [Reference]	0.032	0.050	0.059	0.113
ICER, $/QALY gained^a^	0 [Reference]	Strictly dominated^b^	Strictly dominated^b^	56 876	1 094 349

Abbreviations: QALY, quality-adjusted life-year; ICER, incremental cost-effectiveness ratio.

^a^ ICER is calculated relative to the next least-costly, nondominated strategy.

^b^ Strictly dominated indicates that the strategy resulted in higher costs and fewer QALYs compared with another strategy.

^c^ Extendedly dominated indicates that the strategy resulted in fewer QALYs at a higher cost per QALY gained compared with another strategy.

### Sensitivity Analyses

**Figure 2.**
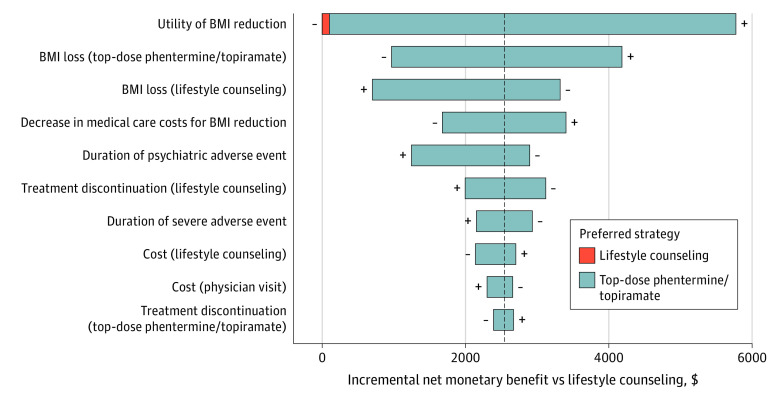
One-Way Sensitivity Analyses Over 5-Year Time Horizon Mid-dose phentermine and topiramate indicates 7.5 mg phentermine and 46 mg topiramate; top-dose phentermine and topiramate, 15 mg phentermine and 92 mg topiramate; BMI, body mass index. Model parameters were independently varied across a set range while all other parameters were held constant at their mean value. The 10 model parameters with the largest effect on the incremental net monetary benefit relative to lifestyle counseling at a willingness-to-pay threshold of $100 000 per quality-adjusted life-year are shown. Parameters with the largest effect are located at the top, and parameters with the smallest effect are shown at the bottom. The incremental net monetary benefit represents the monetary value of an intervention for a given WTP threshold and is calculated as incremental quality-adjusted life-years × willingness-to-pay − incremental costs. An incremental net monetary benefit greater than $0 indicates the strategy is cost-effective compared with lifestyle counseling. The horizontal bars represent the range of incremental net monetary benefit when changing the value of each model parameter, and the color indicates which strategy was preferred. A change in the preferred strategy is shown with a colored vertical bar at the end of the horizontal bar. + and − at the end of the horizontal bars denote that the incremental net monetary benefit was calculated at the maximum and minimum value of the parameter, respectively. A tornado diagram was not generated for a time horizon of 13 months and 2 years, as the preferred strategy in most of the parameter ranges examined was lifestyle counseling.

At 13 months, lifestyle counseling remained the preferred strategy across all parameter ranges in 1-way sensitivity analyses. At 2 years, top-dose phentermine and topiramate became preferred when its cost was at its minimum value and utility of BMI reduction was at its maximum value. Mid-dose phentermine and topiramate was preferred at its minimum cost. At 5 years, top-dose phentermine and topiramate was the preferred strategy across most parameter ranges (Figure 2). Lifestyle therapy was preferred when the utility of BMI reduction was at its minimum value.

**Figure 3.**
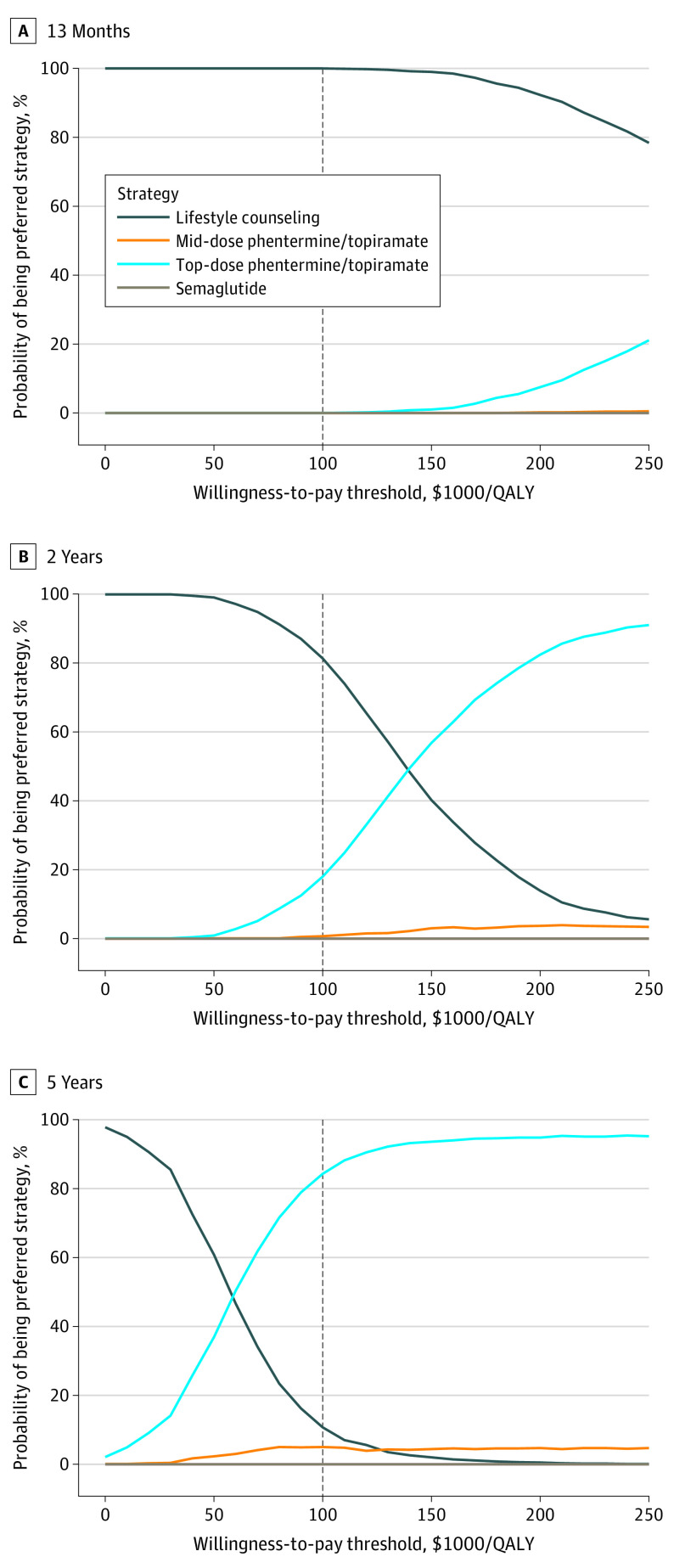
Cost-Effectiveness Acceptability Curves The dashed line indicates the base case willingness-to-pay threshold of $100 000 per quality-adjusted life-year (QALY) gained.

Using a WTP threshold of $100 000 per QALY gained, probabilistic sensitivity analysis estimated lifestyle counseling to be the preferred strategy in 100.0% of 1000 probabilistic iterations at 13 months and 81.3% of 1000 probabilistic iterations at 2 years (Figure 3 and eFigure 5 in Supplement 1). By 5 years, top-dose phentermine and topiramate was the preferred strategy in 84.3% of simulations, followed by lifestyle counseling at 10.7%, and mid-dose phentermine and topiramate at 5.0%.

Due to semaglutide’s effectiveness in maintaining BMI reduction from baseline, we performed a threshold analysis to determine the price reduction needed for it to become the preferred treatment strategy. At 13 months, the monthly cost of semaglutide would need to be reduced by 97.5% (ie, cost $32 per month) for it to become cost-effective (eFigure 6 in Supplement 1). At 2 and 5 years, the monthly cost of semaglutide would need to be reduced 89.4% and 85.2%, respectively, to become the preferred strategy. Changes in mortality rates did not appreciably affect cost-effectiveness results (eTable 8 in Supplement 1).

## Discussion

In this economic evaluation, we used a simulation model to examine the cost-effectiveness of 3 AOMs adjunct to lifestyle counseling to treat adolescents with obesity. AOMs were not estimated to be cost-effective compared with lifestyle counseling alone at 13 months and 2 years. At 5 years, top-dose phentermine and topiramate was projected to be the preferred strategy, with an ICER of $56 876 per QALY gained vs lifestyle counseling. Our results were sensitive to utility of BMI reduction and BMI loss associated with lifestyle counseling and top-dose phentermine and topiramate. Semaglutide resulted in the greatest gain in QALYs but was not projected to be cost-effective due to its high monthly cost, which would need to be reduced more than 85% to become cost-effective.

Several analyses have evaluated the cost-effectiveness of school-based programs,^45,46^ policy changes,^47^ or bariatric surgery^24,48^ for the prevention or treatment of obesity in children and adolescents. However, less is known about the cost-effectiveness of AOMs in this population. To our knowledge, this is the first analysis to compare liraglutide, phentermine and topiramate, and semaglutide for the treatment of obesity in adolescents. Adult studies have generally demonstrated the cost-effectiveness of AOMs over short (ie, ≤5 years)^16,42,49^ and long (ie, ≥30 years)^50,51,52^ time horizons with some exceptions.^53^ Four adult cost-effectiveness analyses included liraglutide, phentermine and topiramate, and semaglutide as comparators.^16,50,51,52^ All models estimated semaglutide to have the greatest gain in QALYs, and 2 models projected phentermine and topiramate to be the cost-effective strategy,^50,52^ similar to our findings.

The goal of obesity treatment in adolescents is to improve quality of life and reduce the risk of future chronic disease.^11^ Physical health benefits with AOMs may not be observed for years, but quality of life improvements may be seen more quickly. Adolescents with obesity experience weight stigma, especially in school settings.^54^ Therefore, short-term analyses may be useful in this school-age population as short-term weight loss was still associated with increased QALYs in our model. With the recent release of guidelines from the AAP, which recommend the use of AOMs for adolescents ages 12 years or older,^7^ our analysis is timely and underscores the need to compare the effectiveness and cost-effectiveness of AOMs.

Though semaglutide was the only strategy estimated to have a BMI reduction relative to baseline at 5 years, its monthly price of nearly $1300 resulted in an ICER well above our WTP threshold. The high cost of AOMs, especially out-of-pocket, is a major barrier that may discourage patients from receiving or adhering to the aggressive treatment that the AAP recommends.^7^ Most state Medicaid plans do not cover AOMs, and private insurance often has strict criteria for authorization.^55,56^ Given that obesity is a chronic disease, mounting AOM costs over time may prevent use of these medications.

### Limitations

The results of our analysis should be considered in the context of several limitations. We did not include all FDA-approved AOMs for adolescents. We excluded orlistat, since it is currently rarely used due to AEs and limited tolerability.^7^ While lifestyle counseling often has high attrition rates, it was included as a comparator because it is the foundational approach for BMI reduction.^7^ Our cost estimate for lifestyle counseling may not have represented the intensity of counseling provided in the adolescent clinical trials, since the number of contact hours in the trials was not reported. We used a limited societal perspective that did not include all indirect costs. We assumed patients receiving no treatment maintained their BMI-for-age percentile and did not incorporate secular trends or individual heterogeneity in BMI trajectories. Our model assumed BMI regain occurred at a constant rate and may not have accounted for nonlinear associations of costs and mortality with BMI. Because model inputs were estimated from multiple sources, sensitivity analyses were used to address input uncertainty and showed that model results remained robust. Due to lack of long-term adolescent data, we extrapolated BMI changes and treatment adherence from adult data and assumed no treatment discontinuation or further BMI reduction after 2 years of treatment. While there is evidence that adults discontinue AOMs within 2 years,^51,57^ we modeled a maximum of 5 years of treatment, since obesity requires long-term treatment.^8^ We may have underestimated the benefit of AOMs, since obesity-related comorbidities may be prevented or delayed in adulthood.^7^ However, due to limited research with long-term follow-up, it is unknown whether AOMs lead to sustained BMI reduction, and it is unclear how comorbidities develop through childhood to adulthood.^7^ Modeled patients were based on participants in clinical trials and were not a nationally representative sample. Future clinical trials should enroll participants who are representative of the US population, including low-income; racial, ethnic, gender, or sexual minority; and other underrepresented patients and should report differential outcomes based on patient subgroups. The ideal follow-up time of future trials is at least 10 to 20 years to capture benefits of disease prevention.

## Conclusions

In this economic evaluation, we projected top-dose phentermine and topiramate adjunct to lifestyle counseling to be cost-effective for the treatment of obesity in US adolescent patients after 5 years. While semaglutide led to the greatest reduction in BMI, it was not cost-effective due to its high cost. Long-term clinical trials are needed to fully understand the safety, efficacy, and cost-effectiveness of AOMs in adolescents.

## Notes

Supplement 1.
eMethods.
**eTable 1.** BMI-Specific All-Cause Annual Mortality Rates Among Males and Females **eTable 2.** Number of Months to Return to Natural BMI Based on Treatment Length **eTable 3.** Relative Change in BMI From Baseline Calibration Targets **eTable 4.** Relative Change in BMI From Baseline in Adult Clinical Trials **eTable 5.** Proportion of Patients Who Continued Treatment in Adult Clinical Trials **eTable 6.** Key Model Assumptions **eTable 7.** Model Output of Relative BMI Change From Baseline **eTable 8.** Cost-Effectiveness Results Over Each Time Horizon Using Non-BMI Specific Life Tables **eFigure 1.** Relative BMI Change From Baseline Over a 5-Year Time Horizon **eFigure 2.** BMI Over a 5-Year Time Horizon **eFigure 3.** Relative BMI Change From Natural BMI Trajectory Over 5-Year Time Horizon **eFigure 4.** Cost-Effectiveness Planes **eFigure 5.** Incremental Cost-Effectiveness Scatterplots **eFigure 6.** Threshold Analysis for Monthly Cost of Semaglutide
eReferences.

Supplement 2.
Data Sharing Statement
