# Supplementary material for: Cost-Effectiveness of Pharmacotherapy for the Treatment of Obesity in Adolescents
Source: JAMA Netw Open. 2023 Aug 31;6(8):e2329178. doi: 10.1001/jamanetworkopen.2023.29178 (PMC10472196; doi:10.1001/jamanetworkopen.2023.29178)
Supplement: Supplement 1. — eMethods. eTable 1. BMI-Specific All-Cause Annual Mortality Rates Among Males and Females eTable 2. Number of Months to Return to Natural BMI Based on Treatment Length eTable 3. Relative Change in BMI From Baseline Calibration Targets eTable 4. Relative Change in BMI From Baseline in Adult Clinical Trials eTable 5. Proportion of Patients Who Continued Treatment in Adult Clinical Trials eTable 6. Key Model Assumptions eTable 7. Model Output of Relative BMI Change From Baseline eTable 8. Cost-Effectiveness Results Over Each Time Horizon Using Non-BMI Specific Life Tables eFigure 1. Relative BMI Change From Baseline Over a 5-Year Time Horizon eFigure 2. BMI Over a 5-Year Time Horizon eFigure 3. Relative BMI Change From Natural BMI Trajectory Over 5-Year Time Horizon eFigure 4. Cost-Effectiveness Planes eFigure 5. Incremental Cost-Effectiveness Scatterplots eFigure 6. Threshold Analysis for Monthly Cost of Semaglutide eReferences. [file jamanetwopen-e2329178-s001.pdf]

Supplemental Online Content

Lim F, Bellows BK, Tan SX, et al. Cost-effectiveness of pharmacotherapy for the treatment of obesity in adolescents. *JAMA Netw Open*. 2023;6(8):e2329178.
doi:10.1001/jamanetworkopen.2023.29178

eMethods.
eTable 1. BMI-Specific All-Cause Annual Mortality Rates Among Males and Females
eTable 2. Number of Months to Return to Natural BMI Based on Treatment Length
eTable 3. Relative Change in BMI From Baseline Calibration Targets
eTable 4. Relative Change in BMI From Baseline in Adult Clinical Trials
eTable 5. Proportion of Patients Who Continued Treatment in Adult Clinical Trials
eTable 6. Key Model Assumptions
eTable 7. Model Output of Relative BMI Change From Baseline
eTable 8. Cost-Effectiveness Results Over Each Time Horizon Using Non-BMI Specific Life Tables
eFigure 1. Relative BMI Change From Baseline Over a 5-Year Time Horizon
eFigure 2. BMI Over a 5-Year Time Horizon
eFigure 3. Relative BMI Change From Natural BMI Trajectory Over 5-Year Time Horizon
eFigure 4. Cost-Effectiveness Planes
eFigure 5. Incremental Cost-Effectiveness Scatterplots
eFigure 6. Threshold Analysis for Monthly Cost of Semaglutide
eReferences.

This supplemental material has been provided by the authors to give readers additional information about their work.

© 2023 Lim F et al. *JAMA Network Open.*

eMethods.

Modeled Time Horizons

We assessed the cost-effectiveness of lifestyle counseling alone and adjunct to antiobesity medications (AOMs) over three different time horizons: 13 months, 2 years, and 5 years. A 13-month time horizon was chosen as it was the most common treatment period across all adolescent clinical trials. The liraglutide study by Kelly et al[1] and the phentermine and topiramate study by Kelly et al[2] were conducted over 56 weeks, or approximately 13 months. The semaglutide trial by Weghuber et al[3] was conducted over 68 weeks, or approximately 16 months. We believed a 13-month time horizon was appropriate for our analysis as it represented the maximum duration for which we have clinical trial data specifically for adolescents using liraglutide, phentermine and topiramate, and semaglutide.

We chose a 2-year time horizon as it represented the maximum duration for which we have adult clinical trial data for liraglutide, phentermine and topiramate, and semaglutide.[4–6] Adult clinical trials reported results after 2 years of treatment which we used to project outcomes after the treatment period of the adolescent trials.

Lastly, we chose a time horizon of 5-years as a high-end value for treatment duration. While there is evidence to suggest that most adult patients discontinue AOMs within 2 years,[7] it is currently unknown if this also applies to adolescents. However, obesity is a chronic disease that requires long-term treatment and management. For this reason, we projected outcomes until 5 years but are hesitant to extrapolate past this time point due to exponentially increasing uncertainty from a lack of published data, especially for adolescents. The American Academy of Pediatrics has noted these significant gaps in research in their clinical practice guideline for evaluation and treatment of obesity in children and adolescents.[8] They state that there is limited research with long-term follow-up which evaluates whether treatment leads to sustained weight reduction. Therefore, we did not project outcomes beyond 5 years due to high uncertainty regarding long-term treatment with AOMs.

Body Mass Index Regain after Treatment Discontinuation

Body mass index **(**BMI, calculated as weight in kilograms divided by height in meters squared) regain after a patient discontinued treatment was modeled from the adolescent clinical trial by Kelly et al[1] which evaluated liraglutide over 56 weeks, or approximately 13 months. It included an additional 26-week follow-up period that began when all patients discontinued treatment. We used the following steps to estimate BMI regain after discontinuation. First, we projected a natural BMI trajectory from baseline for subjects in the trial, assuming they never received any treatment, using the Extended BMI-for-age Growth Charts from the Centers for Disease Control and Prevention (CDC)[9]. This approach is explained in the Methods. Second, we estimated a line of best fit for the observed data during the 26-week period after discontinuation from the liraglutide trial. Third, we assumed a constant rate of BMI gain from the line of best fit to extrapolate beyond the 26-week period to determine how many months it took for BMI to return to the natural BMI trajectory. Using this approach, when treatment in the trial was discontinued at 13 months, it took 17 months for the projected BMI to reach the natural BMI trajectory. Finally, we used this approach to estimate the number of months to return to the natural BMI trajectory, assuming a constant rate of BMI gain, for each month of treatment with liraglutide from the trial. The resulting months to return to the natural BMI trajectory after discontinuation were used for all AOMs in the simulation and are shown in **eTable 2**.

Calibration of BMI Changes

We calibrated the model to reproduce intention-to-treat values for the relative change in BMI from baseline reported in the adolescent clinical trials.[1–3] Calibration was performed using simulated annealing. It was an iterative process that randomly selected values from a set range that represented the maximum BMI reduction on treatment. To determine the suitability of the selected values, we used the chi-square goodness of fit to compare our model output (i.e., the mean BMI change from baseline for all patients) with our calibration target (i.e., the intention-to-treat values shown in **eTable 3**).

Projecting 2-Year and 5-Year BMI Change

Because the treatment period of the adolescent clinical trials ranged between approximately 13 and 16 months,[1–3] we used data from longer term adult clinical trials to project changes in BMI for our 2-year and 5-year time horizons.[4–6,10,11] Lifestyle counseling was modeled from placebo results from the adolescent liraglutide clinical trial by Kelly et al[1] which showed a 0.35% increase in BMI from baseline after 13 months. However, placebo results from the adult clinical trials, which also included lifestyle counseling, reported a reduction in BMI by the end of treatment period. Due to this difference in efficacy between adolescents and adults, we did not use adult data to project BMI changes for lifestyle counseling in adolescents and instead assumed no further weight reduction

© 2023 Lim F et al. *JAMA Network Open.*

occurred after 13 months. Patients continuing to receive lifestyle counseling after 13 months were assumed to maintain the same relative BMI reduction compared to their natural BMI trajectory until the end of the time horizon.

To project changes in BMI with AOMs, we used data from adult clinical trials that reported changes in weight after approximately 1 year and 2 years of treatment.[4–6,10,11] To estimate the BMI at these two time points, we converted the absolute weight in kilograms to BMI by multiplying the weight by the baseline BMI to baseline weight ratio. This method was used in a previously published model for the treatment of obesity in adults that we adapted for our analysis.[12] We then calculated the relative change in BMI from baseline at these two time points to allow for comparison (**eTable 4**). From 1 year to 2 years of treatment on liraglutide, BMI reduction decreased by 27%.[4] From 13 months to 25 months of treatment on mid-dose phentermine and topiramate and top-dose phentermine and topiramate, BMI reduction increased by 19% and 9%, respectively.[5,10] From 16 months to 1 year of treatment on semaglutide, BMI reduction increased by 2%.[6,11] We assumed that the relative change in BMI reduction observed in adults also applied to our adolescent patients over the same time period. Therefore, we adjusted the results of the adolescent clinical trials using the previously mentioned relative changes to estimate the BMI change from baseline after approximately 2 years of treatment (**eTable 3**).

Because the 13-month treatment period for the adolescent liraglutide study by Kelly et al[1] does not align with the 1-year and 2-year results of the adult liraglutide study, we assumed the relative BMI reduction decreased by 27% over 1 full year, from 13 months to 25 months of treatment.

Probability of Treatment Discontinuation

A patient's probability of discontinuing treatment was determined from adolescent clinical trials.[1–3] The liraglutide by Kelly et al[1] and phentermine and topiramate trial by Kelly et al[2] reported the number of patients who completed treatment over 56 weeks, or approximately 13 months. The semaglutide trial by Weghuber et al[3] reported the number of patients who completed treatment over 68 weeks, or approximately 16 months. We determined the proportion of patients who discontinued treatment by subtracting the proportion of patients who completed treatment from 1. This became the probability of discontinuing treatment for the treatment period of the trial and was converted to a monthly probability. Discontinuation of lifestyle counseling was based on placebo results of the adolescent liraglutide study by Kelly et al[1] alone as it was also referenced to model BMI changes for lifestyle counseling.

To project treatment discontinuation beyond the time frame of the adolescent clinical trials, we used data from longer term adult clinical trials which reported the number of patients who completed treatment (**eTable 5**).[4–6,10,11] We used the following steps to calculate the proportion of adolescents who discontinued therapy. First, we calculated the proportion of adults who continued to receive treatment throughout the first year. Second, among those who continued treatment throughout the first year, we calculated the proportion of patients who then continued treatment throughout the second year. Third, we calculated the relative change in the proportion of patients who continued to receive treatment during the second year relative to that of the first year. Lifestyle counseling was based on placebo results of the adult phentermine and topiramate studies by Garvey et al[6] and Gadde et al.[10] Compared with the proportion of patients who completed the first year of treatment, the proportion of patients completing the second year increased by 50% on lifestyle counseling,[4] 3% on liraglutide,[4] 19% on mid-dose phentermine and topiramate,[5,10] 30% on top-dose phentermine and topiramate,[5,10] and 5% on semaglutide.[6,11] Fourth, we assumed this relative increase in the proportion of patients who continued to receive treatment applied to our modeled adolescent patients. We determined the proportion of patients who discontinued treatment by subtracting the proportion of patients who completed treatment from 1 and converted this value into a monthly probability.

Because the 13-month treatment period for the adolescent liraglutide study by Kelly et al[1] does not align with the 1-year and 2-year results of the adult liraglutide study, we assumed the increase in completion for lifestyle counseling and liraglutide occurred over 1 full year, from 13 months to 25 months of treatment.

Adverse Events

Adverse events (AEs) attributable to AOM treatment were incorporated into the model based on results from the adolescent clinical trials.[1–3] We modeled gastrointestinal, psychiatric, and severe AEs as they were the main three types of AEs reported across all studies. Because the actual number of AEs was not consistently reported across all studies, we derived a monthly probability of developing an AE for each AOM based on the reported percentage of patients who experienced the AE. For each type of AE, the percentage of patients on placebo who experienced the AE was subtracted from the percentage of patients on AOM treatment who experienced the AE. This difference was assumed to be the probability of the treatment-related AE over the duration of the clinical trial. We then converted this probability to a monthly value. If the difference was negative, meaning fewer patients

experienced the AE on AOM treatment compared with placebo, we assumed the probability of experiencing the AE with AOM treatment was 0.

Quality-of-Life Adjustments

Increase in utility for a 1-unit reduction in BMI was estimated from a previously published analysis that evaluated the cost-effectiveness of bariatric surgery in adolescents. Bairdain and Samnaliev[13] used the nationally representative Medical Expenditures Panel Survey (MEPS) to estimate the relationship between BMI and health-related quality of life. They reported a utility gain of 0.0042 for a 1-unit decrease in BMI. This estimate was age- and gender- adjusted and specifically generated for MEPS respondents with severe obesity, or a BMI greater than or equal to 34. Therefore, it is an appropriate estimate for our modeled population who also has severe obesity at baseline.

A short-term reduction in utility was applied to patients who experienced an AOM-related gastrointestinal, psychiatric, and severe AE. Due to limited adolescent data, the disutility of a gastrointestinal AE was estimated from utilities of different health states in adult patients with type 2 diabetes.[14] Matza et al[14] reported that the difference in utility for a basic health state and a basic health state with nausea was 0.04. Weghuber et al[3] reported that gastrointestinal AEs in adolescent patients using semaglutide only lasted a median of 2 to 3 days. Therefore, we applied a disutility of 0.04 for 3 days for each gastrointestinal AE in the model.

The disutility associated with a psychiatric AE was estimated from differences in health-related quality of life in teenagers with and without depression.[15] Lynch et al[15] reported that subthreshold depression (e.g., does not meet full diagnostic criteria for depression) was associated with a 0.099 to 0.174 decrease in health-related quality of life compared with no depression. We used a base case value of 0.117. The adolescent clinical trials did not report the severity or duration of psychiatric AEs. Therefore, we assumed the disutility of psychiatric AEs was applied for the same duration as gastrointestinal AEs (i.e., 3 days). Uncertainty in duration was explored in one-way sensitivity analyses. The lower bound was 0 days and the upper bound was 14 days.

We estimated the generic disutility of a severe AE from a previously published cost-effectiveness analysis that assumed the disutility of a serious AE from blood pressure medication use was 0.10 applied for 2 weeks.[16] We applied the same disutility value for the same duration to our modeled patients who experienced a severe AE. Uncertainty in the duration of severe AEs was explored in one-way sensitivity analyses. The lower bound was 0 weeks and the upper bound was 4 weeks.

Healthcare Sector and Limited Societal Perspective

Our analysis was conducted from a healthcare sector perspective, which includes all direct medical costs regardless of payer, and a limited societal perspective, which includes caregiver productivity loss. We incorporated total direct medical care costs for adolescents with obesity which included third-party and out-of-pocket medical expenditures.[17] We assumed a visit to a physician and a session of lifestyle counseling were associated with a 2-hour loss of time from the primary caregiver. This was inclusive of travel time, waiting time, and examination or counseling time. Gastrointestinal and psychiatric AEs were associated with a loss of 48 hours (3 days minus 8 hours per day for sleeping) for the caregiver. Severe AEs were associated with a loss of 224 hours (2 weeks minus 8 hours per day for sleeping) for the caregiver. Duration of psychiatric and severe AEs were varied in one-way sensitivity analyses which subsequently varied the number of hours of productivity loss.

Costs

The cost of lifestyle counseling was estimated from a study that reported the cost of a family-based intervention for pediatric obesity.[18] The cost per child reported in Table 2 from Janicke et al.[18] was $872. This value was inflated to 2022 US dollars using the healthcare component of the Consumer Price Index.[19] The inflated cost was $1,201 which was used as the annual cost of lifestyle counseling. Therefore, the monthly cost of lifestyle counseling in the model was $100.

The family-based intervention in Janicke et al.[18] was administered over 4 months in a rural community with a total of 12 visits and 18 contact hours; however, we believed it was the best estimate for our modeled lifestyle counseling and patient population among all related published literature. Participants were aged 8 to 14 years old with obesity which is just below our model's start age of 15 years. The adolescent AOM clinical trials did not report contact hours, however Kelly et al.[2] reported that lifestyle training sessions were typically between 5 to 15 minutes. Therefore, we believed the lifestyle counseling was below the recommended 26 contact hours which was also the case in Janicke et al.[18] Uncertainty in the cost of lifestyle counseling was explored in one-way and probabilistic sensitivity analyses.

© 2023 Lim F et al. *JAMA Network Open.*

The base case cost of AOMs were estimated from the Center for Medicare and Medicaid Services National Average Drug Acquisition Cost. Upper and lower bounds for AOM costs used in sensitivity analyses were the average wholesale price from Micromedex RED BOOK and the lowest available price from the Department of Veterans Affairs Federal Supply Schedule, respectively.[20,21] While the Federal Supply Schedule is recommended for estimating pharmaceutical costs according to the Second Panel on Cost-Effectiveness in Health and Medicine,[22] we believe the National Average Drug Acquisition Cost is similar to estimates from other sources, such as GoodRX, and represents a reasonable base case cost. The Second Panel does not provide a strong justification for using the Federal Supply Schedule, and they do not necessarily recommend it be used for the costs of other healthcare products. Given the uncertainty and variability of drug prices in the US due to complicated payment and reimbursement systems, we believe it is important to vary AOM costs across a wide, plausible range of values in sensitivity analyses.

The cost of AOM-related severe AEs was estimated form a study that reported the costs of adverse drug events in community hospitals.[23] In Table 3, the adjusted average hospitalization cost for patients with adverse drug events was $10,330. This cost was inflated to 2022 US dollars using the healthcare component of the Consumer Price Index[19] and was modeled as a one-time cost for a severe AE.

We estimated total annual medical care costs for adolescents with severe obesity from a published study that analyzed the Medical Expenditure Panel Survey.[17] Biener et al.[17] reported the annual total expenditures for boys and girls with severe obesity to be $2,640 and $2,566, respectively in Appendix Table 13. These costs were used as the baseline cost of medical care for patients in our model. However, costs were dependent on changes in BMI from baseline. For a 1-unit increase in BMI, annual medical care costs increased by $82 for boys and $77 for girls according to Appendix Table 12. This increase in cost was incorporated in the model for patients who gained BMI relative to their baseline value. Additionally, we assumed that for a 1-unit decrease in BMI, annual medical care costs decreased by the same respective amount for boys and girls. All costs reported were inflated to 2022 US dollars using the healthcare component of the Consumer Price Index.[19]

Evaluating Cost-Effectiveness

An incremental cost-effectiveness ratio (ICER) is a commonly used measure in cost-effectiveness analyses that represents the economic value of a certain intervention compared with another intervention. More specifically, it is the additional cost of a 1 unit increase of an outcome for a specific strategy when compared with another strategy. In our analysis, our outcome of interest is quality-adjusted life years (QALYs). QALYs are used to measure health outcomes and incorporate both the quality and quantity of life lived. The number of years spent in a certain health state is multiplied by its utility (i.e., a value from 0, representing death, to 1, representing perfect value) to calculate the number of QALYs. The calculation of an ICER is shown below:

$$ICER = \frac{\Delta Cost}{\Delta QALYs}$$

When we are comparing multiple interventions simultaneously, interventions are ordered from lowest to highest cost. Strictly dominated interventions have fewer QALYs and higher costs compared with another intervention. These interventions are removed from any consideration and do not have a calculated ICER. Once all strictly dominated interventions are removed, ICERs are calculated for each strategy in comparison with its next lower cost alternative. This means no ICER is calculated for the strategy that has the lowest cost. If a strategy has an ICER greater than the next more costly alternative, it is extendedly dominated which means it results in fewer QALYs with a greater cost per QALY than its comparator. Extendedly dominated strategies are removed from consideration and do not have a calculated ICER. All ICERs are recalculated after the removal of extended dominated strategies. Both strictly dominated and extendedly dominated strategies do not represent an efficient use of sources and are not considered cost-effective when compared with other included strategies.

To determine the cost-effectiveness of an intervention, we compare the intervention's ICER with a willingness-to-pay threshold (WTP) which represents the maximum cost a health system is willing to pay for a benefit in health. If the ICER is below the WTP threshold, the intervention is cost-effective compared with its reference strategy. If the ICER is below the WTP threshold, the intervention is not cost-effective compared with its reference strategy.

Net Monetary Benefits (NMB)

Results from 1-way sensitivity analyses were presented using a net monetary benefit (NMB) framework. NMB represents the monetary value of an intervention for a given willingness-to-pay (WTP) threshold. When

© 2023 Lim F et al. *JAMA Network Open.*

compared with a reference strategy, incremental net monetary benefit (INMB) is the incremental effectiveness, or quality-adjusted life years (QALYs), of a strategy multiplied by the WTP threshold and subtracted by its incremental costs.

$$INMB = \Delta QALYs * WTP\ Threshold - \Delta Costs$$

When the INMB > 0, the strategy is cost-effective compared with the reference strategy at the given WTP threshold. The greater the INMB, the more cost-effective the strategy is when compared with the reference strategy. Therefore, when multiple strategies are being compared simultaneously, the strategy with the overall highest INMB is considered the most cost-effective strategy.

A tornado diagram is often used to visualize the results from 1-way sensitivity analyses. The parameter that had the greatest effect on INMB is located at the top, while the parameter with the smallest effect is located at the bottom. Horizontal bars show the range of INMB values when parameters are varied from their minimum to maximum value. A change in the preferred strategy at the specified WTP is shown with a colored vertical bar.

Bounds and Standard Error for Probability of Treatment Discontinuation

Bounds for probability of treatment discontinuation were calculated from 95% confidence intervals using the formula shown below where *p* is the proportion of patients discontinuing treatment and *n* is the sample size.

$$95\%\ CI = p \pm 1.96\sqrt{\frac{p(1-p)}{n}}$$

The standard error (SE) of the probability of discontinuing treatment was calculated using the formula shown below where *p* is the proportion of patients discontinuing treatment and *n* is the sample size.

$$SE = \sqrt{\frac{p(1-p)}{n}}$$

© 2023 Lim F et al. *JAMA Network Open.*

eTable 1. BMI-Specific All-Cause Annual Mortality Rates Among Males and Females.

**Males**														
Age / BMI	30	31	32	33	34	35	36	37	38	39	40	41	42	43
15	0.00067	0.00082	0.00098	0.00113	0.00127	0.00139	0.00150	0.00162	0.00176	0.00196	0.00224	0.00264	0.00319	0.00391
16	0.00079	0.00094	0.00110	0.00125	0.00139	0.00151	0.00163	0.00174	0.00189	0.00208	0.00236	0.00277	0.00332	0.00404
17	0.0093	0.00108	0.00123	0.00139	0.00152	0.00165	0.00176	0.00188	0.00202	0.00222	0.00250	0.00290	0.00345	0.00417
18	0.00111	0.00121	0.00132	0.00146	0.00161	0.00176	0.00194	0.00215	0.00238	0.00266	0.00286	0.00315	0.00340	0.00375
19	0.00104	0.00113	0.00124	0.00137	0.00150	0.00164	0.00181	0.00200	0.00220	0.00246	0.00265	0.00291	0.00314	0.00345
**Females**														
Age / BMI	30	31	32	33	34	35	36	37	38	39	40	41	42	43
15	0.00040	0.00043	0.00046	0.00050	0.00054	0.00058	0.00063	0.00068	0.00074	0.00081	0.00088	0.00097	0.00107	0.00118
16	0.00044	0.00047	0.00050	0.00054	0.00058	0.00062	0.00067	0.00072	0.00078	0.00085	0.00092	0.00101	0.00111	0.00122
17	0.00048	0.00051	0.00055	0.00058	0.00062	0.00066	0.00071	0.00076	0.00082	0.00089	0.00097	0.00105	0.00115	0.00126
18	0.00053	0.00057	0.00061	0.00065	0.00069	0.00075	0.00080	0.00085	0.00090	0.00097	0.00103	0.00109	0.00116	0.00125
19	0.00052	0.00055	0.00059	0.00064	0.00067	0.00073	0.00078	0.00083	0.00088	0.00094	0.00100	0.00106	0.00112	0.00121

Abbreviations: BMI, body mass index

© 2023 Lim F et al. *JAMA Network Open.*

eTable 2. Number of Months to Return to Natural BMI Based on Treatment Length.

Months on Treatment	Months to Return to Natural History BMI
1	5
2	8
3	9
4	11
5	13
6	14
7	14
8	15
9	15
10	15
11	16
12	16
13+	17

Abbreviations: BMI, body mass index

© 2023 Lim F et al. *JAMA Network Open.*

eTable 3. Relative Change in BMI From Baseline Calibration Targets

Strategy	Relative Change in BMI (%) [Range]	Source
Lifestyle counseling		
13 months	0.4 [-2.1 to 1.4][a]	Kelly et al,[1] 2020
Liraglutide		
13 months	-4.3 [-6.0 to -2.6][a]	Kelly et al,[1] 2020
25 months	-3.2 [-4.4 to -1.9]	Kelly et al,[1] 2020; Astrup et al,[4] 2012
Mid-dose phentermine and topiramate		
13 months	-4.8 [-7.3 to -2.2][a]	Kelly et al,[2] 2022
25 months	-5.7 [-8.7 to -2.7]	Kelly et al,[2] 2022; Gadde et al,[10] 2011; Garvey et al,[5] 2012
Top-dose phentermine and topiramate		
13 months	-7.1 [-9.1 to -5.1][a]	Kelly et al,[2] 2022
25 months	-7.8 [-9.9 to -5.6]	Kelly et al,[2] 2022; Gadde et al,[10] 2011; Garvey et al,[5] 2012
Semaglutide		
16 months	-16.1 [-19.6 to -12.6][a]	Weghuber et al,[3] 2022
24 months	-16.4 [-20.0 to -12.8]	Weghuber et al,[3] 2022; Wilding et al,[11] 2021; Garvey et al,[6] 2022

Abbreviations: BMI, body mass index

[a] Intention-to-treat value reported in the corresponding adolescent clinical trial.

© 2023 Lim F et al. *JAMA Network Open.*

eTable 4. Relative Change in BMI From Baseline in Adult Clinical Trials.

Strategy	Relative Change in BMI (%)	Source
Liraglutide		
1 year	-8.5	Astrup et al,[4] 2012
2 years	-6.2	
Mid-dose phentermine and topiramate		
13 months	-7.8	Gadde et al,[10] 2011
25 months	-9.3	Garvey et al,[5] 2012
Top-dose phentermine and topiramate		
13 months	-9.8	Gadde et al,[10] 2011
25 months	-10.7	Garvey et al,[5] 2012
Semaglutide		
16 months	-14.9	Wilding et al,[11] 2021
24 months	-15.2	Garvey et al,[6] 2022

Abbreviations: BMI, body mass index

© 2023 Lim F et al. *JAMA Network Open.*

eTable 5. Proportion of Patients Who Continued Treatment in Adult Clinical Trials.

Strategy	Proportion of Patients	Source
Lifestyle counseling		
0-1 years	0.63	Astrup et al,[4] 2012
1-2nd years[a]	0.76	
Liraglutide		
0-1 years	0.70	Astrup et al,[4] 2012
1-2nd years[a]	0.72	
Mid-dose phentermine/topiramate		
0-13 months	0.69	Gadde et al,[10] 2011
14-25 months[a]	0.82	Garvey et al,[5] 2012
Top-dose phentermine/topiramate		
0-13 months	0.64	Gadde et al,[10] 2011
14-25 months[a]	0.83	Garvey et al,[5] 2012
Semaglutide		
0-16 months	0.83	Wilding et al,[11] 2021
17-24 months[a]	0.87	Garvey et al,[6] 2022

[a] Proportion of patients who continued treatment during this period was conditional upon completing treatment throughout the first period.

© 2023 Lim F et al. *JAMA Network Open.*

eTable 6. Key Model Assumptions.

Model Component	Assumptions	Source
Model Type	• Microsimulation model	
Population	• 100,000 hypothetical adolescents based on the average participant characteristics across all adolescent clinical trials<br>• Age 15 years, initial BMI of 37, 58% female	Kelly et al,[1] 2020;<br>Kelly et al,[2] 2022;<br>Weghuber et al,[3] 2022;
Interventions	• Lifestyle counseling<br>• Lifestyle counseling adjunct to liraglutide<br>• Lifestyle counseling adjunct to mid-dose phentermine and topiramate<br>• Lifestyle counseling adjunct to top-dose phentermine and topiramate<br>• Lifestyle counseling adjunct to semaglutide	
Time Horizon	• 13 months<br>• 2 years<br>• 5 years	
Perspective	• Healthcare sector perspective<br>• Limited societal perspective	
Treatment Adherence	• Estimated from adolescent clinical trials for the duration of the trial<br>• Beyond the treatment period of adolescent clinical trials, estimated from 2-year adult clinical trials<br>• Assumed all patients receiving treatment after 2 years continued to receive treatment until 5 years	Kelly et al,[1] 2020;<br>Kelly et al,[2] 2022;<br>Weghuber et al,[3] 2022;<br>Astrup et al,[4] 2012;<br>Gadde et al,[10] 2011;<br>Garvey et al,[5] 2012;<br>Wilding et al,[11] 2021;<br>Garvey et al,[6] 2022
Natural BMI trajectory with no treatment	• Annual increase in BMI of 1.1<br>• Assumed no change in initial BMI-for-age percentile after 5 years based on Extended BMI-for-age Growth Charts	Growth Charts – CDC Extended BMI-for-Age Growth Charts[9], 2022
BMI change with receiving treatment	• Calibrated model to replicate intention-to-treat values for relative change in BMI from baseline reported in adolescent clinical trials<br>• Beyond the treatment period of adolescent clinical trials, estimated BMI changes using 2-year adult data<br>• Assumed the same relative change in BMI from 1 year to 2 years of treatments in adult patients applied to modeled adolescent patients<br>• After 2 years, assumed the relative difference in current BMI compared with natural BMI trajectory remained constant until 5 years	Kelly et al,[1] 2020;<br>Kelly et al,[2] 2022;<br>Weghuber et al,[3] 2022;<br>Astrup et al,[4] 2012;<br>Gadde et al,[10] 2011;<br>Garvey et al,[5] 2012;<br>Wilding et al,[11] 2021;<br>Garvey et al,[6] 2022

© 2023 Lim F et al. *JAMA Network Open.*

Model Component	Assumptions	Source
BMI regain after treatment discontinuation	• Patients who discontinued treatment regained BMI at an increased rate until returning to their natural BMI trajectory<br>• Rate of regain was dependent on number of months of treatment<br>• Estimated from the 26-week period of treatment discontinuation in the adolescent liraglutide trial	Kelly et al,[1] 2020
Adverse Events	• Included AOM-related gastrointestinal, psychiatric, and severe AEs for the duration of the adolescent clinical trials	Kelly et al,[1] 2020;<br>Kelly et al,[2] 2022;<br>Weghuber et al,[3] 2022
Obesity-related Medical Care Costs	• Included annual medical care costs associated with severe obesity<br>• Costs increased for a 1-unit gain of BMI and decreased for a 1-unit reduction of BMI	Biener et al,[17] 2020
Comorbidities	• Not incorporated in the model due to limited research with long-term follow up that evaluates how comorbidities develop throughout childhood into adulthood according to the American Academy of Pediatrics	Hampl et al,[8] 2023

Abbreviations: BMI, body mass index; AOM, antiobesity medication

© 2023 Lim F et al. *JAMA Network Open.*

eTable 7. Model Output of Relative Change in BMI From Baseline.

Strategy	Relative Change in BMI (%)
Lifestyle counseling	
13 months	0.4
Liraglutide	
13 months	-4.3
25 months	-3.2
Mid-dose phentermine and topiramate	
13 months	-4.8
25 months	-5.7
Top-dose phentermine and topiramate	
13 months	-7.1
25 months	-7.8
Semaglutide	
16 months	-16.1
24 months	-16.4

Abbreviations: BMI, body mass index

© 2023 Lim F et al. *JAMA Network Open.*

eTable 8. Cost-Effectiveness Results Over Each Time Horizon Using Non-BMI Specific Life Tables.

	Lifestyle counseling	Liraglutide	Mid-dose phentermine and topiramate	Top-dose phentermine and topiramate	Semaglutide
**13 Months**					
Costs, mean ($)	5,054	20,828	7,087	7,215	21,617
Incremental costs, mean ($)	REF	15,774	2,033	2,161	16,562
QALY, mean	0.715	0.718	0.719	0.721	0.726
Incremental QALYs, mean	REF	0.003	0.005	0.007	0.011
ICER ($/QALY gained)[a]	REF	Strictly dominated[b]	Extendedly dominated[c]	322,753	3,048,470
**2 Years**					
Costs, mean ($)	9,053	34,672	11,869	11,650	37,309
Incremental costs, mean ($)	REF	25,619	2,817	2,597	28,256
QALY, mean	1.299	1.308	1.313	1.317	1.333
Incremental QALYs, mean	REF	0.010	0.014	0.019	0.034
ICER ($/QALY gained)[a]	REF	Strictly dominated[b]	Strictly dominated[b]	139,906	1,627,728
**5 Years**					
Costs, mean ($)	21,790	74,879	26,446	25,128	84,140
Incremental costs, mean ($)	REF	53,089	4,655	3,337	62,350
QALY, mean	3.075	3.106	3.124	3.133	3.184
Incremental QALYs, mean	REF	0.031	0.048	0.058	0.109
ICER ($/QALY gained)[a]	REF	Strictly dominated[b]	Strictly dominated[b]	57,950	1,149,782

Abbreviations: QALY, quality-adjusted life year; ICER, incremental cost-effectiveness ratio

© 2023 Lim F et al. *JAMA Network Open.*

eFigure 1. Relative BMI Change From Baseline Over a 5-Year Time Horizon.

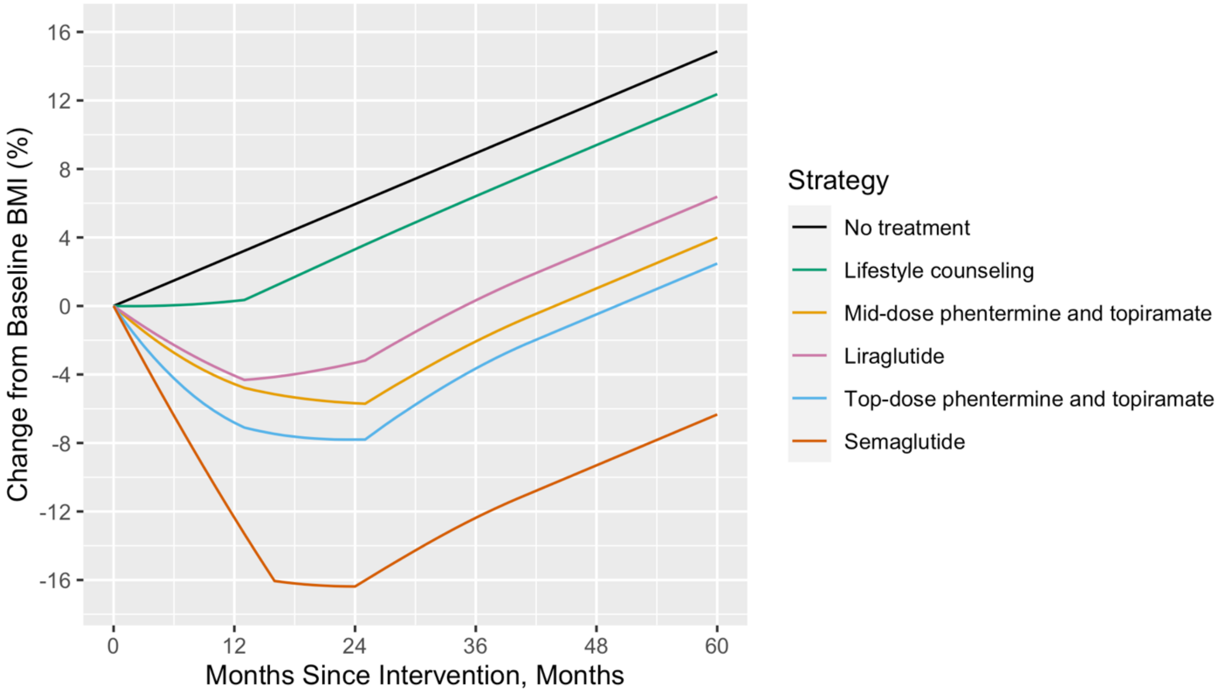

Abbreviations: BMI, body mass index

© 2023 Lim F et al. *JAMA Network Open.*

eFigure 2. BMI Over a 5-Year Time Horizon.

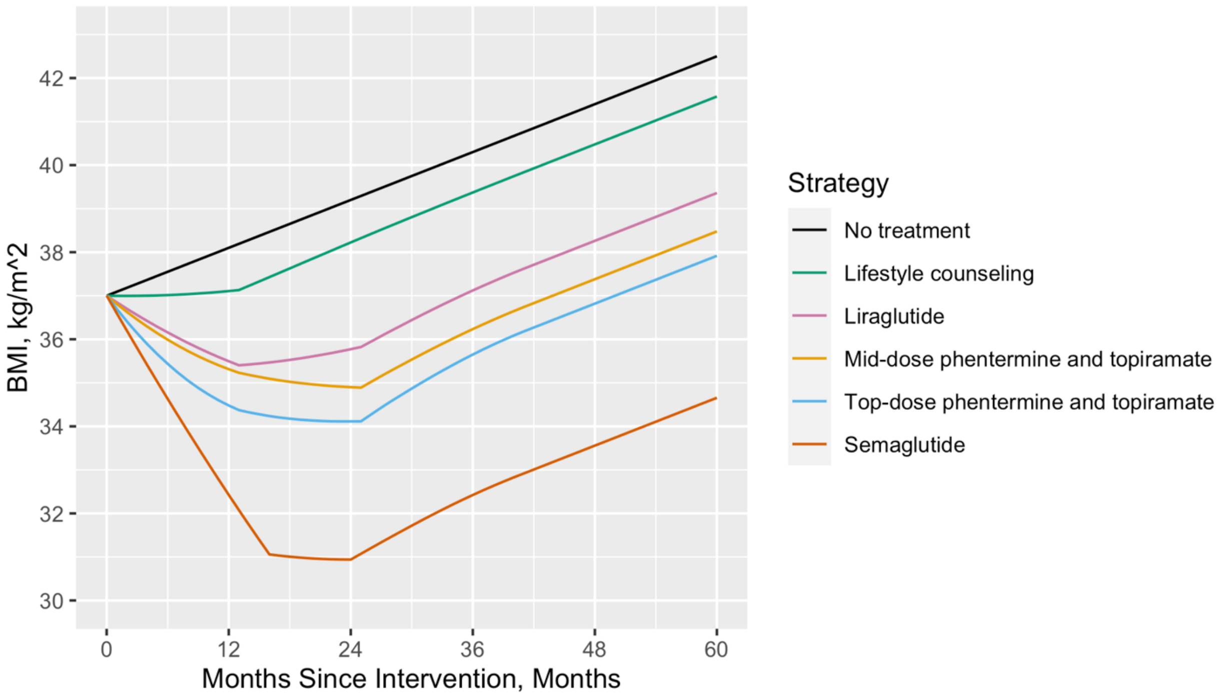

Abbreviations: BMI, body mass index

© 2023 Lim F et al. *JAMA Network Open.*

eFigure 3. Relative BMI Change From Natural BMI Trajectory Over 5-Year Time Horizon.

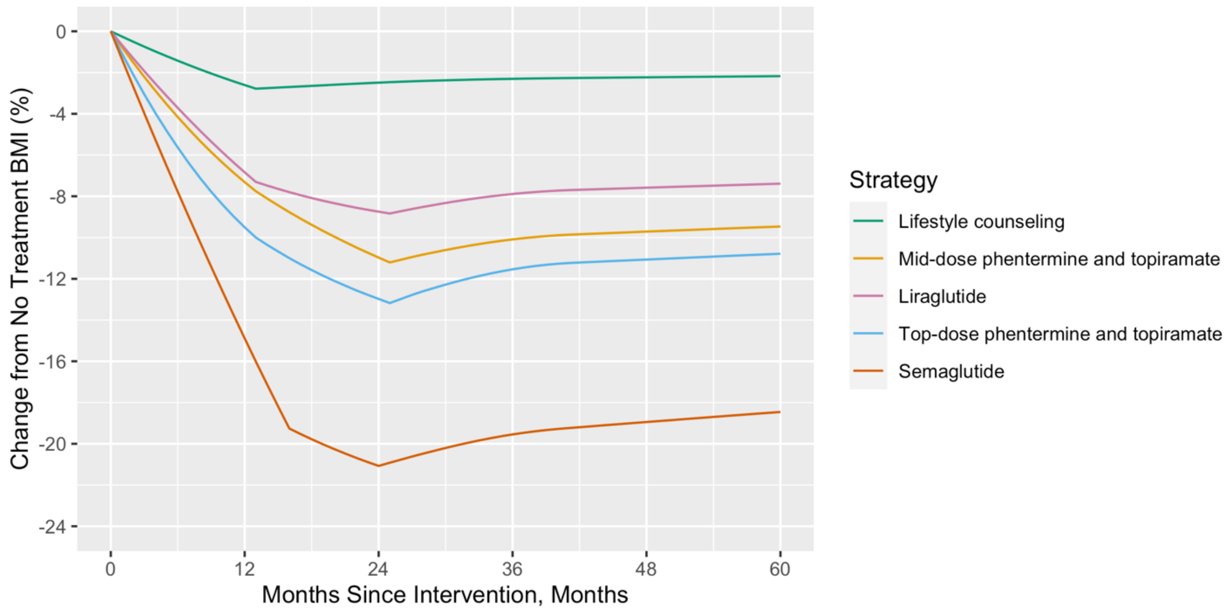

Abbreviations: BMI, body mass index

© 2023 Lim F et al. *JAMA Network Open.*

eFigure 4. Cost-Effectiveness Planes.

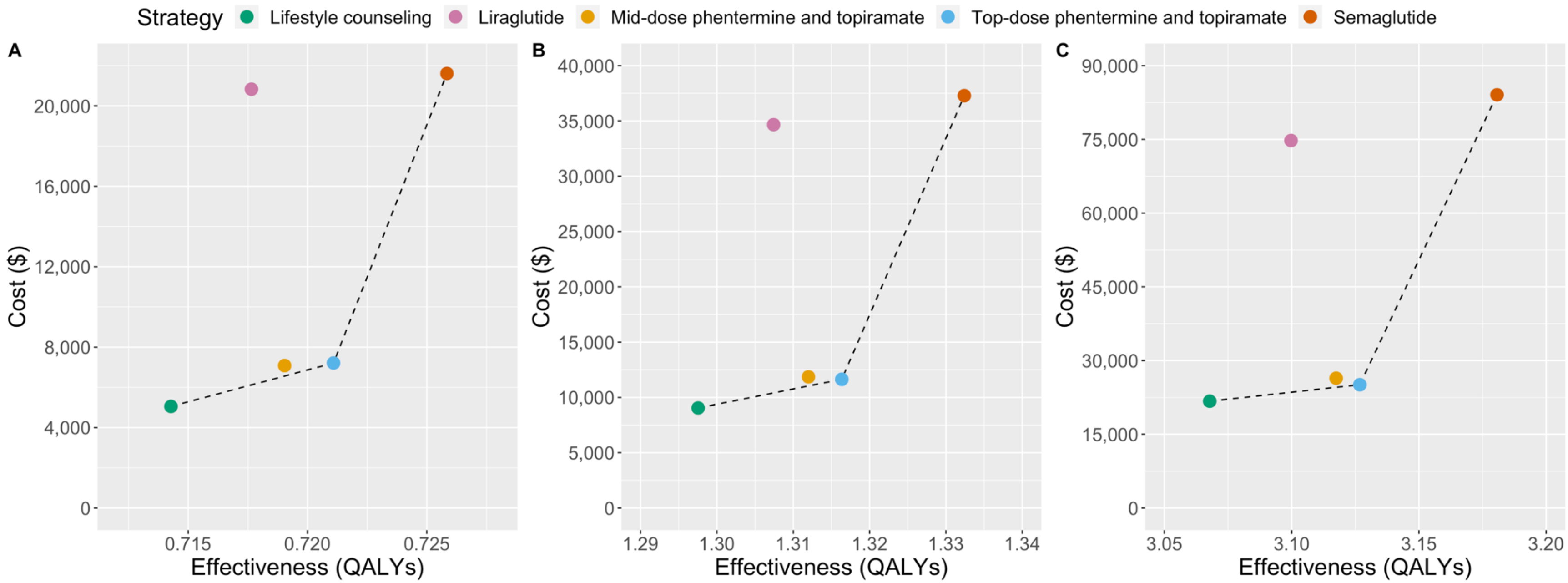

Abbreviations: QALYS, quality-adjusted life years

The figure shows the cost-effectiveness planes after **A)** 13 months, **B)** 2 years, and **C)** 5 years. Dashed lines indicate the efficiency frontier. Any strategy that is above the efficiency frontier is not cost-effective when compared with the other strategies that exist on the efficiency frontier.

© 2023 Lim F et al. *JAMA Network Open.*

eFigure 5. Incremental Cost-Effectiveness Scatterplots.

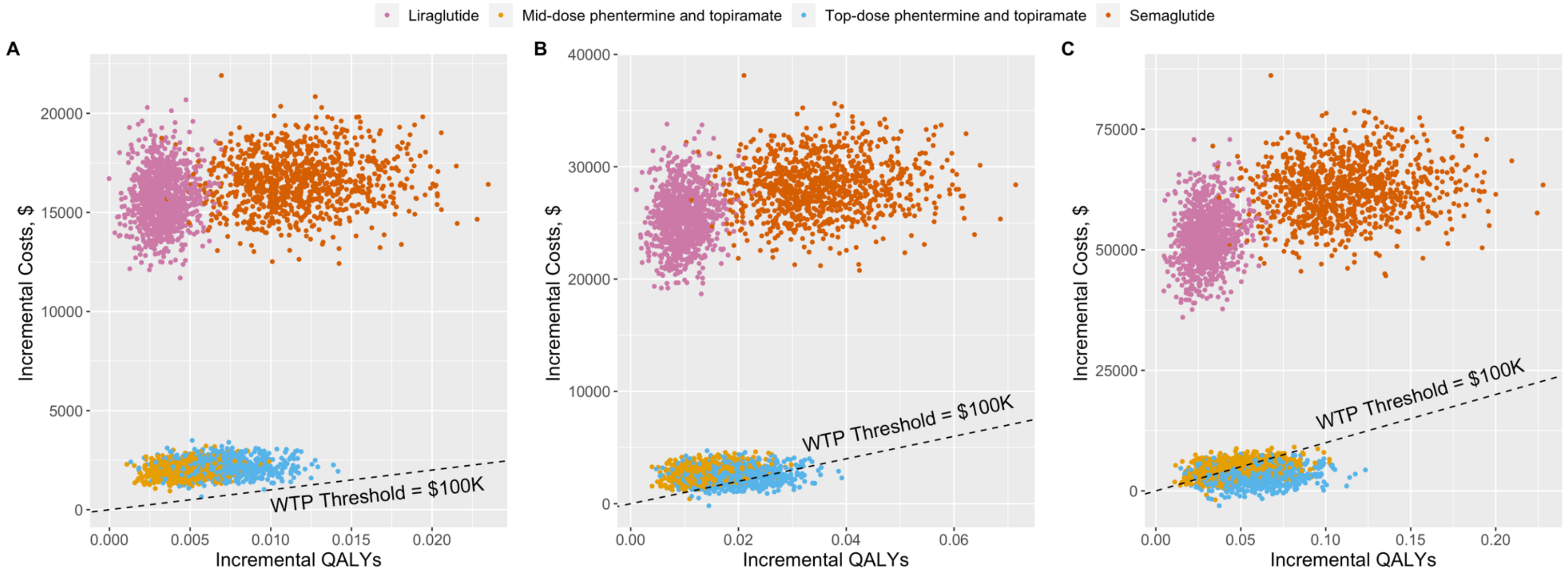

Abbreviations: QALYs, quality-adjusted life years

The figure shows the incremental costs and QALYs relative to lifestyle counseling for 1,000 iterations of our model during our probability sensitivity analysis. The dashed line denotes our willingness-to-pay threshold of $100,000 per QALY gained. Any simulation that exists below the dashed line indicates that the strategy is cost-effective relative to lifestyle counseling. **Panel A** shows results over a time horizon of 13 months. **Panel B** shows results over a time horizon of 2 years. **Panel C** shows results over a time horizon of 5 years.

© 2023 Lim F et al. *JAMA Network Open.*

eFigure 6. Threshold Analysis for Monthly Cost of Semaglutide.

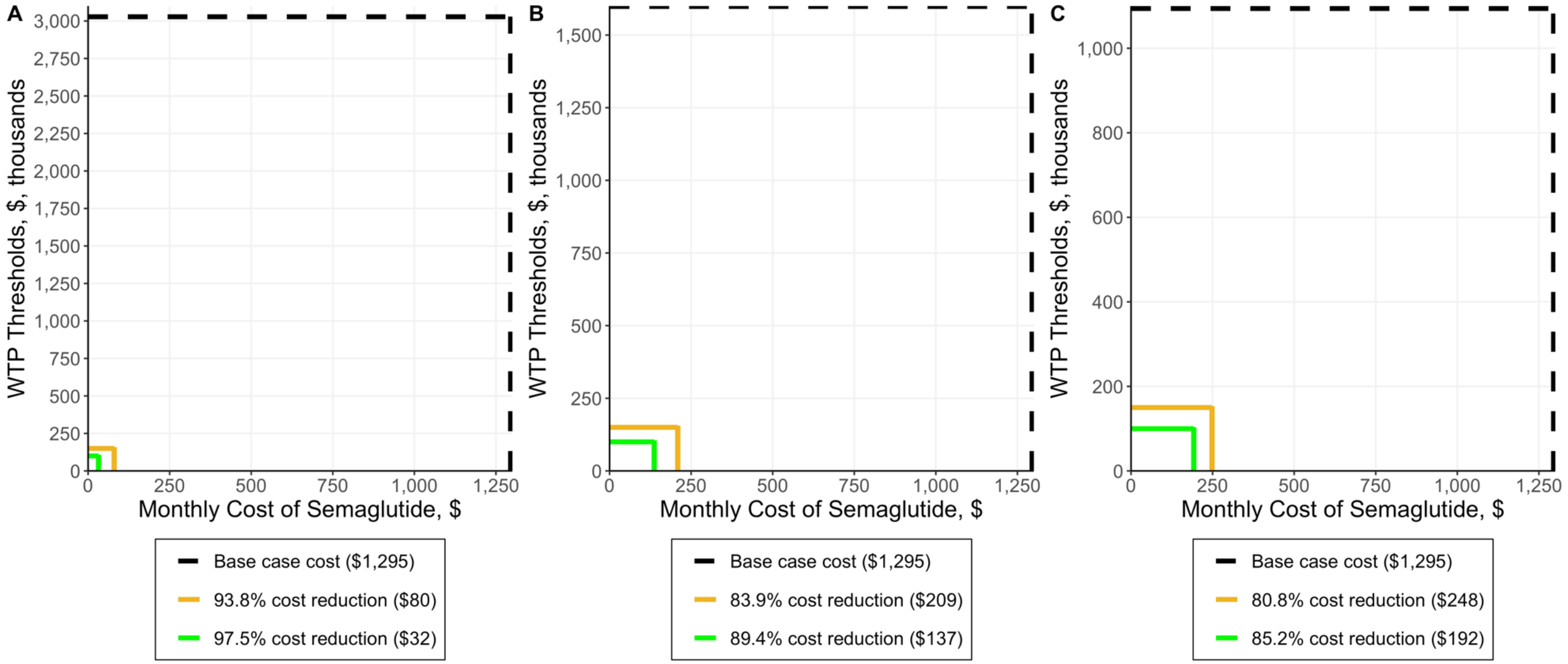

Abbreviations: WTP, willingness-to-pay

The figure shows the percent reduction in monthly cost of semaglutide needed for semaglutide to be the preferred strategy using a willingness-to-pay threshold of $100,000 per quality-adjusted life year (QALY) gained. A black dotted line indicates the base case annual cost of semaglutide and its corresponding incremental cost-effectiveness ratio value. A yellow line indicates the monthly cost of semaglutide required for it to have an ICER of $150,000 per QALY gained. A green line indicates the monthly cost of semaglutide required for it to have an ICER of $100,000 per QALY gained. **Panel A** shows results over a 13-month time horizon. **Panel B** shows results over a 2-year time horizon. **Panel C** shows results over a 5-year time horizon.

© 2023 Lim F et al. *JAMA Network Open.*

eReferences

1. Kelly AS, Auerbach P, Barrientos-Perez M, et al. A Randomized, Controlled Trial of Liraglutide for Adolescents with Obesity. *N Engl J Med*. 2020;382(22):2117-2128. doi:10.1056/NEJMoa1916038

2. Kelly AS, Bensignor MO, Hsia DS, et al. Phentermine/Topiramate for the Treatment of Adolescent Obesity. *NEJM Evid*. 2022;1(6):EVIDoa2200014. doi:10.1056/EVIDoa2200014

3. Weghuber D, Barrett T, Barrientos-Pérez M, et al. Once-Weekly Semaglutide in Adolescents with Obesity. *N Engl J Med*. Published online November 2, 2022. doi:10.1056/NEJMoa2208601

4. Astrup A, Carraro R, Finer N, et al. Safety, tolerability and sustained weight loss over 2 years with the once-daily human GLP-1 analog, liraglutide. *Int J Obes 2005*. 2012;36(6):843-854. doi:10.1038/ijo.2011.158

5. Garvey WT, Ryan DH, Look M, et al. Two-year sustained weight loss and metabolic benefits with controlled-release phentermine/topiramate in obese and overweight adults (SEQUEL): a randomized, placebo-controlled, phase 3 extension study. *Am J Clin Nutr*. 2012;95(2):297-308. doi:10.3945/ajcn.111.024927

6. Garvey WT, Batterham RL, Bhatta M, et al. Two-year effects of semaglutide in adults with overweight or obesity: the STEP 5 trial. *Nat Med*. 2022;28(10):2083-2091. doi:10.1038/s41591-022-02026-4

7. Ganguly R, Tian Y, Kong SX, et al. Persistence of newer anti-obesity medications in a real-world setting. *Diabetes Res Clin Pract*. 2018;143:348-356. doi:10.1016/j.diabres.2018.07.017

8. Hampl SE, Hassink SG, Skinner AC, et al. Clinical Practice Guideline for the Evaluation and Treatment of Children and Adolescents With Obesity. *Pediatrics*. Published online January 9, 2023:e2022060640. doi:10.1542/peds.2022-060640

9. Growth Charts - CDC Extended BMI-for-Age Growth Charts. Published December 21, 2022. Accessed February 17, 2023. https://www.cdc.gov/growthcharts/extended-bmi.htm

10. Gadde KM, Allison DB, Ryan DH, et al. Effects of low-dose, controlled-release, phentermine plus topiramate combination on weight and associated comorbidities in overweight and obese adults (CONQUER): a randomised, placebo-controlled, phase 3 trial. *Lancet Lond Engl*. 2011;377(9774):1341-1352. doi:10.1016/S0140-6736(11)60205-5

11. Wilding JPH, Batterham RL, Calanna S, et al. Once-Weekly Semaglutide in Adults with Overweight or Obesity. *N Engl J Med*. 2021;384(11):989-1002. doi:10.1056/NEJMoa2032183

12. Lee M, Lauren BN, Zhan T, et al. The cost-effectiveness of pharmacotherapy and lifestyle intervention in the treatment of obesity. *Obes Sci Pract*. 2019;6(2):162-170. doi:10.1002/osp4.390

13. Bairdain S, Samnaliev M, Bairdain S, Samnaliev M. Cost-effectiveness of Adolescent Bariatric Surgery. *Cureus*. 2015;7(2). doi:10.7759/cureus.248

14. Matza LS, Boye KS, Yurgin N, et al. Utilities and disutilities for type 2 diabetes treatment-related attributes. *Qual Life Res*. 2007;16(7):1251-1265. doi:10.1007/s11136-007-9226-0

15. Lynch FL, Dickerson JF, Feeny DH, Clarke GN, MacMillan AL. Measuring Health-related Quality of Life in Teens With and Without Depression. *Med Care*. 2016;54(12):1089-1097.

16. Bress AP, Bellows BK, King JB, et al. Cost-Effectiveness of Intensive versus Standard Blood-Pressure Control. *N Engl J Med*. 2017;377(8):745-755. doi:10.1056/NEJMsa1616035

© 2023 Lim F et al. *JAMA Network Open.*

17. Biener AI, Cawley J, Meyerhoefer C. The medical care costs of obesity and severe obesity in youth: An instrumental variables approach. *Health Econ*. 2020;29(5):624-639. doi:10.1002/hec.4007

18. Janicke DM, Sallinen BJ, Perri MG, Lutes LD, Silverstein JH, Brumback B. Comparison of Program Costs for Parent-Only and Family-Based Interventions for Pediatric Obesity in Medically Underserved Rural Settings. *J Rural Health*. 2009;25(3):326-330. doi:10.1111/j.1748-0361.2009.00238.x

19. Bureau of Economic Analysis. Price Indexes for Personal Consumption Expenditures by Type of Product, Table 2.4.4U. Accessed April 8, 2022. https://apps.bea.gov/iTable/iTable.cfm?reqid=19&step=2#reqid=19&step=2&isuri=1&1921=underlying

20. Merative™ Micromedex® RED BOOK® (electronic version). Merative, Ann Arbor, Michigan, USA. Accessed November 7, 2022. Available at: https://www.micromedexsolutions.com/

21. US Department of Veterans Affairs. Office of Procurement, Acquisitions and Logistics (OPAL): pharmaceutical prices. Accessed November 15, 2022. https://www.va.gov/opal/nac/fss/pharmPrices.asp

22. Neumann PJ, Ganiats TG, Russell LB, Sanders GD, Siegel JE, eds. Second Panel on Cost-Effectiveness in Health and Medicine. In: *Cost-Effectiveness in Health and Medicine*. Oxford University Press; 2016:0. doi:10.1093/acprof:oso/9780190492939.002.0007

23. Hug BL, Keohane C, Seger DL, Yoon C, Bates DW. The costs of adverse drug events in community hospitals. *Jt Comm J Qual Patient Saf*. 2012;38(3):120-126. doi:10.1016/s1553-7250(12)38016-1

© 2023 Lim F et al. *JAMA Network Open.*
