# Supplementary material for: Cost-Effectiveness of Pharmacotherapy for the Treatment of Obesity in Adolescents
Source: JAMA Netw Open. 2023 Aug 31;6(8):e2329178. doi: 10.1001/jamanetworkopen.2023.29178 (PMC10472196; doi:10.1001/jamanetworkopen.2023.29178)
Supplement: Supplement 2. — Data Sharing Statement [file jamanetwopen-e2329178-s002.pdf]

Data Sharing Statement

Lim. Cost-Effectiveness of Pharmacotherapy for the Treatment of Obesity in Adolescents. *JAMA Netw Open*. Published August 31, 2023. doi:10.1001/jamanetworkopen.2023.29178

Data
Data available: No

Additional Information
Explanation for why data not available: All data used in the model are available from published literature and publicly available data sources that are cited in our manuscript.
